# Supplementary material for: Predicting visual function by interpreting a neuronal wiring diagram
Source: Nature. 2024 Oct 2;634(8032):113–23. doi: 10.1038/s41586-024-07953-5 (PMC11446822; doi:10.1038/s41586-024-07953-5)
Supplement: Supplementary file 6 — CRF and ERF predictions for individual TmY4 and TmY9 cells. Analogous to Supplementary Data 3, but for TmY target types. Shown are the top four monosynaptic pathways, the strongest pathway passing through each of the top ten intermediary types (ranking from Extended Data Fig. 7), and the trisynaptic pathway Tm1–TmY–Dm3–TmY (see the section entitled Prediction of spatial normalization). [file 41586_2024_7953_MOESM6_ESM.zip › DataS4/TmY9qΓèÑ/720575940646661155.pdf]

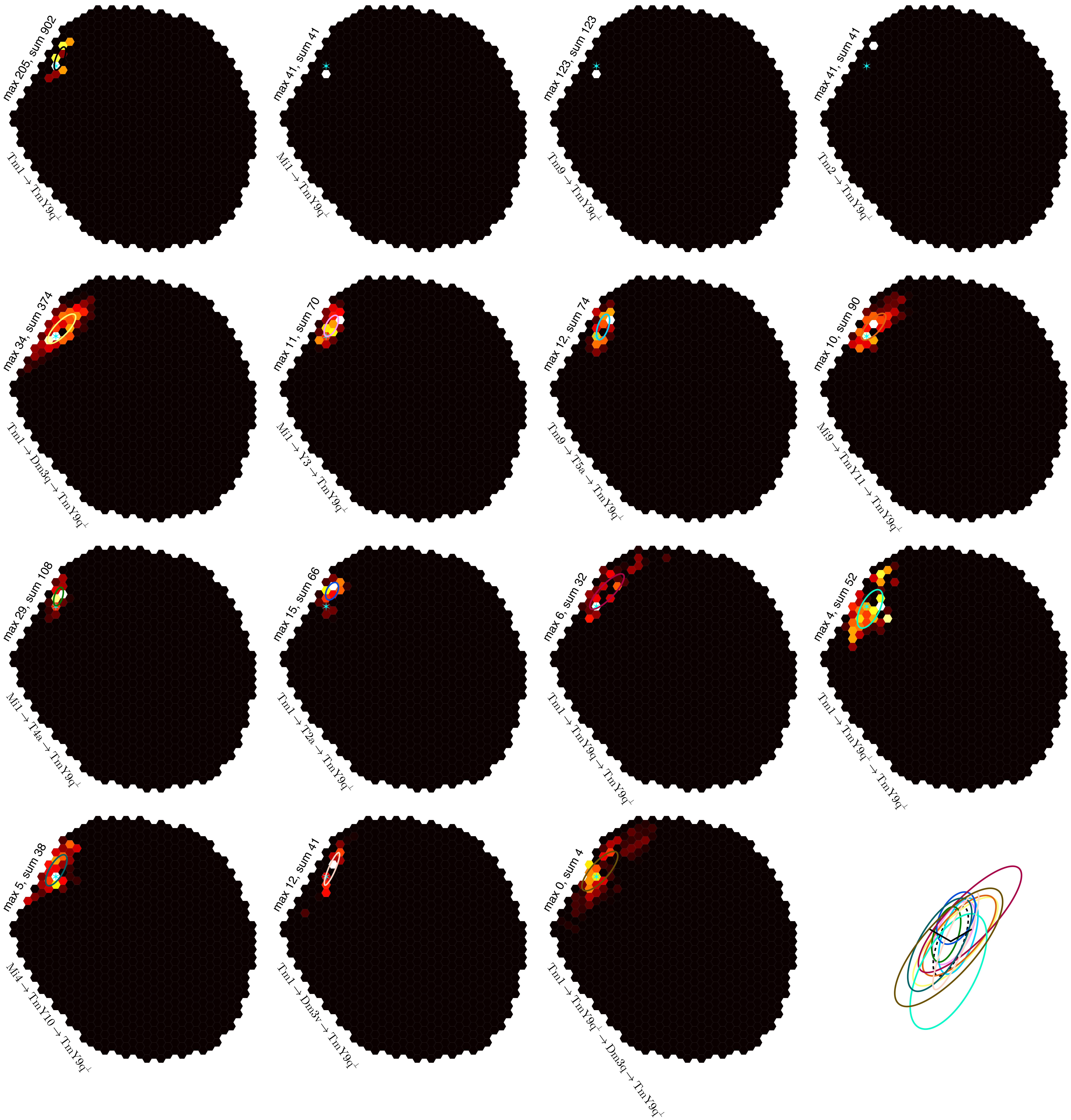
max 205, sum 902
Tm1 → TmY9q⊥
max 41, sum 41
Mi1 → TmY9q⊥
max 123, sum 123
Tm9 → TmY9q⊥
max 41, sum 41
Tm2 → TmY9q⊥
max 34, sum 374
Tm1 → Dm3q → TmY9q⊥
max 11, sum 70
Mi1 → Y3 → TmY9q⊥
max 12, sum 74
Tm9 → T5a → TmY9q⊥
max 10, sum 90
Mi9 → TmY11 → TmY9q⊥
max 29, sum 108
Mi1 → T4a → TmY9q⊥
max 15, sum 66
Tm1 → T2a → TmY9q⊥
max 6, sum 32
Tm1 → TmY9q → TmY9q⊥
max 4, sum 52
Tm1 → TmY9q⊥ → TmY9q⊥
max 5, sum 38
Mi4 → TmY10 → TmY9q⊥
max 12, sum 41
Tm1 → Dm3v → TmY9q⊥
max 0, sum 4
Tm1 → TmY9q⊥ → Dm3q → TmY9q⊥
